# Does the contralateral healthy ankle of patient with ipsilateral mechanical lateral ankle laxity show greater lateral ankle laxity? Evaluation of the anterior talofibular ligament by stress ultrasonography

**DOI:** 10.1186/s12891-022-05838-0

**Published:** 2022-09-30

**Authors:** Takuji Yokoe, Takuya Tajima, Shuichi Kawagoe, Nami Yamaguchi, Yudai Morita, Etsuo Chosa

**Affiliations:** Division of Orthopaedic Surgery, Department of Medicine of Sensory and Motor Organs, Faculty of Medicine, University of Miyazaki, 5200, 889-1692 Kihara, Kiyotake, Miyazaki Japan

**Keywords:** Anterior talofibular ligament, Lateral ankle laxity, Chronic lateral ankle laxity, Ultrasonography, Side difference

## Abstract

**Background:**

A number of studies have evaluated risk factors for lateral ankle sprain (LAS) or chronic lateral ankle instability (CLAI). However, the definitive risk factors for LAS or CLAI remain controversial. The purpose of this study was to evaluate whether the contralateral healthy ankles of subjects with ipsilateral mechanical lateral ankle laxity (group I) show greater lateral ankle laxity in comparison to the healthy ankles of bilateral healthy controls (group B).

**Methods:**

From March 2020, anterior talofibular ligament (ATFL) lengths of young adult volunteers were cross-sectionally measured in non-stress and stress positions using a previously reported stress ultrasonography (US) procedure. The ATFL ratio (the ratio of stress ATFL/non-stress ATFL length) was calculated as an indicator of lateral ankle laxity. The manual anterior drawer test (ADT) was also performed. The US findings of healthy ankles from groups I and B were compared.

**Results:**

A total of 154 subjects in group B (mean age, 24.5 ± 2.8 years; male/female, 84/70) and 40 subjects in group I (mean age, 24.4 ± 2.3 years; male/female, 26/14) were included in the study. There was no significant difference in the ADT between the groups. There were no significant differences in the non-stress ATFL length (19.4 ± 1.8 vs. 19.3 ± 1.9, p = 0.84), stress ATFL length (20.8 ± 1.8 vs. 20.9 ± 1.9, p = 0.66), length change (1.5 ± 0.6 vs. 1.6 ± 0.6, p = 0.12) and ATFL ratio (1.08 ± 0.03 vs. 1.08 ± 0.03, p = 0.13) between the groups.

**Conclusion:**

No significant difference was detected between the contralateral healthy ankles of subjects with ipsilateral mechanical lateral ankle laxity and those of bilateral healthy controls.

## Background

Lateral ankle sprain (LAS) is among the most frequent musculoskeletal injuries in the general population, as well as in athletes [1]. Unfortunately, a large proportion of patients who suffered from LAS will develop to chronic lateral ankle instability (CLAI), which is associated with the subsequent development of ankle osteoarthritis [2–4]. Although CLAI has been studied by many researchers in the orthopaedic field, the risk factors for CLAI remain controversial. Doherty et al. reported that the inability to perform a drop-landing or drop-vertical jump within two weeks after primary LAS was associated with the occurrence of CLAI [5]. Pourkazemi et al. found that younger age (odds ratio [OR], 8.41) and primary ankle sprain (OR, 8.23) were independent predictors of recurrent ankle sprains [6]. Recently, Lee et al. reported that the contralateral healthy ankles of patients with CLAI showed poor postural stability and neuromuscular control [7], and recommended rehabilitation of the unaffected ankles to prevent future ankle sprains. This study indicates that the unaffected ankles of subjects with ipsilateral mechanical lateral ankle laxity would be more prone to LAS when compared with bilateral healthy controls. Are the healthy contralateral ankles of subjects with ipsilateral lateral ankle laxity more likely to have greater native laxity when compared with those of bilateral healthy controls? Regarding studies of the anterior cruciate ligament (ACL) injury, it was reported that patients with an ipsilateral ACL injury showed significantly increased anterior and internal rotation of the contralateral healthy knee joint in comparison to healthy volunteers [8, 9]. To our knowledge, no studies have investigated whether the contralateral healthy ankles of subjects with ipsilateral lateral ankle laxity show greater laxity in comparison to healthy controls.

Stress ultrasonography (US) has been reported to be a reliable and useful tool for evaluating lateral ankle laxity [10, 11]. It has been reported that the anterior talofibular ligament (ATFL) ratio, which is defined as a ratio of stress ATFL length to non-stress ATFL length, is a useful parameter to assess lateral ankle laxity by stress US [10, 12, 13]. However, no previous studies have evaluated whether the contralateral healthy ankles of subjects with ipsilateral mechanical lateral ankle laxity can be used as reference to assess lateral ankle laxity for the diagnosis of CLAI or the evaluation of surgical outcomes.

The purpose of this study was to evaluate whether the contralateral healthy ankles of subjects with ipsilateral mechanical lateral ankle laxity show greater lateral ankle laxity when compared with the healthy ankles of bilateral healthy controls. It was hypothesized that the contralateral healthy ankles of subjects with ipsilateral mechanical lateral ankle laxity would have greater lateral ankle laxity in comparison to bilateral healthy controls.

## Methods

This retrospective study was designed to investigate whether the contralateral side of the ankle can be used as a reference when evaluating mechanical lateral ankle laxity. All procedures were in accordance with the ethical standards of the responsible committee on human experimentation (institutional and national) and with the Declaration of Helsinki of 1975, as revised in 2013. US findings of the ATFL were cross-sectionally collected from March 2020, and the normative data regarding ATFL in the young general population have been reported previously [12]. In the present study, data obtained from March 2020 to May 2022 were retrospectively evaluated.

After receiving approval from an institutional review board, potential healthy volunteers of 20–35 years of age were recruited via an advertisement in a single institute. All individuals received written information and written informed consent was obtained before participating in this study. The exclusion criteria of this study were as follows: episodes of giving way of the ankle, primary LAS within twelve months of the time of recruitment, previous surgical treatment of the foot or ankle, bilateral mechanical lateral ankle laxity, acute foot and ankle pain at the time of recruitment, osteoarthritis of the ankle, inflammatory arthritis such as rheumatoid arthritis, generalized joint laxity and Ehlers-Danlos or Marfan syndrome. Additionally, considering the recall bias of a history of LAS, ankles were excluded from the analysis when the absence of ATFL, lax and wavy ATFL, or avulsion fracture of the distal fibula was detected by US [14, 15].

A total of 257 subjects (514 ankles) were screened. Sixty-three subjects were excluded for the following reasons: acute LAS (n = 4), foot and ankle pain (n = 2), history of fracture surgery (n = 2), bilateral mechanical lateral ankle laxity (n = 26) and generalized joint laxity (n = 29). According to the study by Yokoe et al. [12], the normative value of the ATFL ratio were 1.07 ± 0.04 in men and 1.09 ± 0.04 in women. The ATFL ratio was defined as the ratio of stress ATFL length to the non-stress ATFL length [16]. In the present study, the mechanical lateral ankle laxity was defined as ATFL ratio > 1.15 for men and > 1.17 for women, as these reference standards were twice the magnitude of each standard deviation. Therefore, among the 194 subjects included in the study, a total of 154 subjects without bilateral mechanical lateral ankle laxity (group B) and 40 subjects with ipsilateral mechanical lateral ankle laxity (group I) were included in the study. The subject characteristics of the two groups are shown in Table 1. There were no significant differences in the baseline characteristics of the two groups. The foot size was defined as the length from the longest toe to the tip of the heel that was measured with a tape measure in the standing position.

**Table 1:** Participant characteristics

Variables	Group B (n = 154)	Group I (n = 40)	P value
Age, year	24.5 ± 2.8	24.4 ± 2.3	0.74
Sex, male/female	84/70	26/14	0.23
Height, cm	166.0 ± 9.2	167.4 ± 8.0	0.40
Weight, kg	60.1 ± 11.5	59.5 ± 10.1	0.92
Body mass index	21.7 ± 2.8	21.3 ± 2.6	0.43
Foot size, cm	24.5 ± 1.7	24.9 ± 1.6	0.11
Beighton score	1.4 ± 1.3	1.5 ± 1.3	0.61

Data are shown as means ± standard deviations unless otherwise indicated.

Group B, subjects with bilateral healthy ankles

Group I, subjects with ipsilateral mechanical lateral ankle laxity

The assessment of GJL and the manual anterior drawer test [ADT] were performed prior to the US examination. GJL was assessed using the Beighton score [17]. The Beighton score has been demonstrated to be reliable and valid [18]. The Beighton score consists of five objective measurements of joint mobility, four of which are measured bilaterally. One point is given when each joint meets the criteria with a score of 0–9. A score of ≥ 5 was defined as GJL according to the recommendation by the international Ehlers-Danlos syndrome Consortium [19]. The ADT was performed by a certified orthopaedic surgeon. US images of the ankle were obtained in the non-stress position (resting position) and the stress position (manual maximal internal rotational position) according to the previously reported method [12, 13]. US evaluations were performed by a certified orthopaedic surgeon who was experienced in this US technique and blinded to participant data. The inter-rater and intra-rater reliability of this US procedure have been previously confirmed [12, 13].

### Manual ADT

ADT of the ankle was performed with the patient in the supine position. The knee joint was flexed, and the ankle joint was sustained in 10–15° plantar flexion. While grasping the heel of the examined ankle with one hand and stabilizing the distal tibia with the other hand, the ankle was drawn until no further movement was recognized. The patient was instructed to relax before the examiner performed the procedure. The results were classified into three grades: Grade 1, a stable joint; Grade 2, partially unstable; Grade 3, completely unstable [10].

### US evaluation of the ATFL

US examinations were performed with an ALOKA ARIETTA 850 US apparatus (HITACHI, Tokyo, Japan) using a linear probe (L64 probe, 18 − 5 MHz). The ATFL lengths were evaluated in two positions: the resting position (non-stress ATFL) and the manual maximal internal rotation (stress ATFL). Non-stress ATFL images were obtained first. The subject was in a sitting position with one leg hanging from the edge of the examination table (resting position), with 10–15° internal rotation of the lower leg. The transducer was placed over the ATFL and was parallel to the sole of the foot. The subject was then instructed to relax his or her ankle muscles with the ankle joint in 10–20° plantar flexion. The ATFL length was measured as the linear distance from the origin to the insertion of the ATFL. The origin and insertion points of the ATFL were identified as bony landmarks during the acquisition of the US images to ensure standardization of the ATFL in a previously reported manner [20]. Thereafter, the stress ATFL image was obtained. The subject was first instructed to position themselves in the resting position, and the examiner manually applied maximal internal rotation and varus talar tilt stress to the ankle. The ATFL length was measured as the linear distance from the origin to the insertion of the ATFL, in the same manner as that for non-stress ATFL images. The anterolateral aspect of the lateral malleolus was identified as the ATFL origin, and the peak of the talus was used as the insertion point. The peak of the talus also represents the anterior aspect of the lateral talar articular cartilage and the lateral neck of the talus. These bony landmarks can be identified as hyperechogenic points [21], and were confirmed to ensure that the talar insertion was consistently selected at a reference point across images. The mean value of the three measurements of the ATFL length was used. Based on the obtained data, the ATFL ratio was calculated.

### Statistical analyses

All statistical analyses were performed using the SAS software (JMP Pro, ver. 15.2.0; SAS Institute, Cary, NC, USA). The results were reported as mean values with 95% confidence intervals (CIs). The Shapiro-Wilk test was performed to confirm the normal distribution of the data. When the data showed a normal distribution, Student t test was conducted to compare continuous data. Otherwise, the Mann-Whitney U test was performed. The chi-square test was used to compare categorial data. Paired Wilcoxon signed rank tests were performed to compare the bilateral ankles. P values < 0.05 were considered statistically significant.

## Results

The comparison of the ADT and stress US findings between the healthy ankles in groups I and B is shown in Table 2. There was no significant difference in the ADT results of the two groups. There were no significant differences between the two groups in the non-stress ATFL length (19.4 ± 1.8 [95% CI, 19.2–19.6] vs. 19.3 ± 1.9 [95% CI, 18.7–19.9], p = 0.84), stress ATFL length (20.8 ± 1.8 [95% CI, 20.6–21.0] vs. 20.9 ± 1.9 [95% CI, 20.3–21.5], p = 0.66), length change (1.5 ± 0.6 [95% CI, 1.4–1.5] vs. 1.6 ± 0.6 [95% CI, 1.4–1.8], p = 0.12) and ATFL ratio (1.08 ± 0.03 [95% CI, 1.07–1.08] vs. 1.08 ± 0.03 [95% CI, 1.07–1.09], p = 0.13).

**Table 2:** Comparison of the results of groups B and I

Variables	Group B (n = 154)	Group I (n = 40)	P value	
Ankle laterality, right/left	154/154	23/17	0.48	
Anterior drawer test			0.08	
grade 1, n (%)	250 (81.2)	37 (92.5)		
grade 2, n (%)	58 (18.8)	3 (7.5)		
Ultrasonographic findings				
nonstress ATFL length, mm				
all ankles	19.4 ± 1.8 (19.2–19.6)	19.3 ± 1.9 (18.7–19.9)	0.84	
	range, 15.7–24.6	range, 16.4–23.1		
right ankles	19.3 ± 1.8 (19.0-19.6)	19.0 ± 1.7 (18.2–19.6)	0.39	
	range, 15.8–23.3	range, 16.5–22.2		
left ankles	19.4 ± 1.8 (19.2–19.7)	19.9 ± 2.2 (18.8–20.9)	0.39	
	range, 15.7–24.6	range, 16.4–23.1		
stress ATFL length, mm				
all ankles	20.8 ± 1.8 (20.6–21.0)	20.9 ± 1.9 (20.3–21.5)	0.66	
	range, 16.4–26.3	range, 17.5–24.9		
right ankles	20.7 ± 1.8 (20.4–21.0)	20.5 ± 1.8 (19.7–21.2)	0.71	
	range, 16.7–26.0	range, 17.5–23.6		
left ankles	20.9 ± 2.0 (20.6–21.3)	21.5 ± 2.0 (20.5–22.5)	0.22	
	range, 16.7–26.0	range, 17.5–23.6		
length change, mm				
all ankles	1.5 ± 0.6 (1.4–1.5)	1.6 ± 0.6 (1.4–1.8)	0.12	
	range, 0.4–3.1	range, 0.5–2.9		
right ankles	1.4 ± 0.6 (1.3–1.5)	1.6 ± 0.5 (1.3–1.8)	0.16	
	range, 0.4–3.1	range, 0.6–2.6		
left ankles	1.5 ± 0.6 (1.4–1.6)	1.6 ± 0.7 (1.3-2.0)	0.40	
	range, 0.5–3.1	range, 0.6–2.9		
ATFL ratio				
all ankles	1.08 ± 0.03 (1.07–1.08)	1.08 ± 0.03 (1.07–1.09)	0.13	
	range, 1.02–1.17	range, 1.02–1.16		
right ankles	1.07 ± 0.03 (1.07–1.08)	1.08 ± 0.03 (1.07–1.10)	0.15	
	range, 1.02–1.17	range, 1.03–1.13		
left ankles	1.08 ± 0.03 (1.07–1.08)	1.09 ± 0.04 (1.07–1.10)	0.45	
	range, 1.03–1.16	range, 1.02–1.16		

Data are shown as means ± standard deviations unless otherwise indicated.

The number in the parenthesis shows 95% confidence interval.

Bilateral ankles were included in group B.

Group B, subjects with bilateral healthy ankles; Group I, subjects with ipsilateral mechanical lateral ankle laxity

ATFL ratio, stress ATFL length / nonstress ATFL length.

ADT, anterior drawer test; ATFL, anterior talofibular ligament.

## Discussion

The most important finding of the present study was that no significant difference was identified between the contralateral healthy ankles of subjects with ipsilateral mechanical lateral ankle laxity and those of bilateral healthy controls, which was contrary to the study hypothesis. The results of this study suggest that the healthy ankles of subjects with ipsilateral lateral ankle laxity can be used as a reference when evaluating lateral ankle laxity on stress US.

Many authors have investigated causative factors that may contribute to the increased occurrence of CLAI [5, 6, 22, 23]. It was reported that dynamic balance deficits, which were assessed by the star excursion balance test, may be associated with an increased risk of CLAI [23]. Raeder et al. recently reported that the delayed initiation of functional therapy after a LAS (> 4 weeks) and females over 41 years of age were predictors of the subsequent development of CLAI [24]. McKay et al. reported that > 50% of patients who incurred ankle sprains did not visit hospitals [25], which suggests that poor understanding of the clinical significance of LAS would be prevalent among patients. Although education and improved understanding of the clinical significance of LAS or CLAI by the general population are mandatory to prevent the progression of LAS to CLAI [26], the identification of risk factors for CLAI is crucial for clinicians when treating or counseling patients who suffer from LAS.

Whether native lateral ankle laxity affects the development of CLAI remains unclear and has not been well studied. With regard to ACL injury, it was reported that patients with an ipsilateral ACL injury showed significantly increased anterior and internal rotation of the contralateral healthy knee joint compared with healthy volunteers [8, 9]. The authors of these studies discussed the influence of native knee rotational characteristics on noncontact ACL injury. Regarding recurrent shoulder instability, Cheng et al. found that rugby players with unstable shoulders have significantly higher shoulder translation in their uninjured shoulder than healthy players [27]. In the present study, individuals who had contralateral mechanical lateral ankle laxity did not show significantly greater elongation of the ATFL than bilateral healthy controls, indicating that native lateral ankle laxity may not be a risk factor of future CLAI. Several studies have shown that females have greater native lateral ankle laxity than males [12, 28]. However, it remains controversial whether female subjects have an increased risk of LAS or CLAI. Waterman et al. reported that the incidence rates of ankle sprain in men and women were 2.20 and 2.10 per 1000 person-years, respectively (NS) [29]. Similar findings were observed in young male and female athletes [30]. Namely, the only native lateral ankle laxity itself may not determine the risk of LAS or CLAI. Regarding risk factors for LAS or CLAI, multiple factors should be considered, such as age, patient activity level, ankle range of motion, generalized hypermobility and type of occupation or sport. Well-designed prospective studies will be required to clarify the influence of native lateral ankle laxity on the incidence of LAS or CLAI.

Clinicians may use the lateral ankle laxity of the contralateral non-injured ankle as a reference when evaluating ipsilateral mechanical lateral ankle laxity. The present study suggested that the healthy ankles of subjects with ipsilateral lateral ankle laxity can be used as a reference when evaluating lateral ankle laxity on stress US. Specific studies are lacking to investigate the native lateral ankle laxity of patients with ipsilateral mechanical ankle laxity, thus, these study findings will aid clinicians in evaluating patients with CLAI. However, the accurate evaluation of the injury or laxity of the ankle lateral ligaments still remains challenging for clinicians [31, 32]. A recent meta-analysis reported that the sensitivity and specificity of the manual ADT were 54% (95% CI, 35-71%) and 87% (95% CI, 63-96%), respectively. The authors recommended a combination of palpation and ADT to make a correct diagnosis of the ATFL injury [33]. Therefore, the findings of the present study may not be generalizable to the evaluation of the lateral ankle laxity by other procedures, such as clinical examinations or stress radiography. Future studies will be needed to evaluate native lateral ankle laxity in patients with mechanical ankle laxity using various kinds of imaging modalities, with continued advancement in clinical tests.

There were several limitations to the present study. First, the subtalar instability was not evaluated in this study. Therefore, it remains unclear whether there was a significant difference in subtalar joint instability between groups B and I. Second, we did not consider the presence of functional ankle instability using such as Cumberland Ankle Instability Tool or Ankle Instability Instrument [34], which may have affected the results. Third, individuals with GJL were not included in the study. Finally, the US evaluation was influenced by the skill of the examiner and the US apparatus.

## Conclusion

The contralateral healthy ankles of subjects with ipsilateral mechanical lateral ankle laxity did not show greater native lateral ankle laxity in comparison to bilateral healthy controls. The study results suggest that healthy ankles of subjects with ipsilateral lateral ankle laxity can be used as a reference when evaluating lateral ankle laxity on stress US.

## Data Availability

All data are available from the corresponding author upon reasonable request.

## List of Abbreviations

LAS: lateral ankle sprain
CLAI: chronic lateral ankle instability
ATFL: anterior talofibular ligament
ADT: anterior drawer test
US: ultrasonography OR, odds ratio
ACL: anterior cruciate ligament
CI: confidence interval
